# Supplementary material for: A New Fit Assessment Framework for Common Factor Models Using Generalized Residuals
Source: Psychometrika. 2025 Aug 7;90(4):1419–44. doi: 10.1017/psy.2025.10037 (PMC12660002; doi:10.1017/psy.2025.10037)
Supplement: Sung et al. supplementary material [file S0033312325100379sup001.zip › supplementary document.pdf]

Supplementary Document for

"A New Fit Assessment Framework for Common Factor Models Using Generalized Residuals"

Contents

A Asymptotic Normality of Transformed Residuals **1**

A.1 Assumptions 1

A.2 Original Residuals $\boldsymbol{e}_\iota$ 1

A.3 Transformed Residuals $\boldsymbol{e}_\varphi$ 4

A.4 Common Factor Models 5

B Empirical Data Analysis Using the Simple Estimator **8**

References **11**

A Asymptotic Normality of Transformed Residuals

A.1 Assumptions

The following assumptions are made for the latent variable measurement model (with likelihood function expressed by Equation 2).

(A1) *The maximum likelihood (ML) estimator (i.e., Equation 5) is $\sqrt{n}$-consistent. In particular,*

$$\sqrt{n}(\hat{\boldsymbol{\xi}} - \boldsymbol{\xi}) = \boldsymbol{\mathcal{I}}^{-1}(\boldsymbol{\xi}) \frac{1}{\sqrt{n}} \sum_{i=1}^{n} \nabla_{\boldsymbol{\xi}} \log f(\mathbf{Y}_i; \boldsymbol{\xi})^\top + o_p(1), \tag{S1}$$

in which the information matrix $\boldsymbol{\mathcal{I}}(\boldsymbol{\xi}) = \mathbb{E}[\nabla_{\boldsymbol{\xi}} \log f(\mathbf{Y}_i; \boldsymbol{\xi})^\top \nabla_{\boldsymbol{\xi}} \log f(\mathbf{Y}_i; \boldsymbol{\xi})] = -\mathbb{E}[\nabla^2_{\boldsymbol{\xi}\boldsymbol{\xi}} \log f(\mathbf{Y}_i; \boldsymbol{\xi})]$ is positive definite, and $\nabla^2_{\boldsymbol{\xi}\boldsymbol{\xi}} \log f$ denotes the $q \times q$ Hessian matrix of the log-likelihood.

(A2) *The vector of functions $\mathbf{H}(\boldsymbol{y}; \boldsymbol{\xi}_1)$ is squared integrable for all $\boldsymbol{\xi}_1$, i.e., $\int \|\mathbf{H}(\boldsymbol{y}; \boldsymbol{\xi}_1)\|^2 f(\boldsymbol{y}; \boldsymbol{\xi}) d\boldsymbol{y} < \infty$ for all $\boldsymbol{\xi}_1$. There exists an $\omega > 0$ and the associated $\mathcal{F}_\omega = \{\boldsymbol{\xi}_1 : \|\boldsymbol{\xi}_1 - \boldsymbol{\xi}\| \le \omega\}$ such that for all $\boldsymbol{y} \in \mathcal{R}^m$ and $\boldsymbol{\xi}_1, \boldsymbol{\xi}_2 \in \mathcal{F}_\omega$,*

$$\|\mathbf{H}(\boldsymbol{y}; \boldsymbol{\xi}_1) - \mathbf{H}(\boldsymbol{y}; \boldsymbol{\xi}_2)\| \le b(\boldsymbol{y}) \|\boldsymbol{\xi}_1 - \boldsymbol{\xi}_2\|, \tag{S2}$$

in which $\|\cdot\|$ denotes the ℓ_2-norm of a vector. Furthermore, the function $b(\boldsymbol{y})$ in Equation S2 is squared integrable, i.e., $\int b(\boldsymbol{y})^2 f(\boldsymbol{y}; \boldsymbol{\xi}) d\boldsymbol{y} < \infty$.

(A3) *As $\omega \downarrow 0$,*

$$\sup_{\boldsymbol{\xi}_1 \in \mathcal{F}_\omega} \left\| \int \mathbf{H}(\boldsymbol{y}; \boldsymbol{\xi}_1) \Big[f(\boldsymbol{y}; \boldsymbol{\xi}_1) - f(\boldsymbol{y}; \boldsymbol{\xi}) - \nabla_{\boldsymbol{\xi}} \log f(\boldsymbol{y}; \boldsymbol{\xi}) (\boldsymbol{\xi}_1 - \boldsymbol{\xi}) f(\boldsymbol{y}; \boldsymbol{\xi}) \Big] d\boldsymbol{y} \right\| = o(\omega). \tag{S3}$$

A.2 Original Residuals $\boldsymbol{e}_\iota$

We first establish asymptotic normality for the original residuals $\boldsymbol{e}_\iota = \hat{\boldsymbol{\eta}}(\hat{\boldsymbol{\xi}}) - \boldsymbol{\eta}(\hat{\boldsymbol{\xi}})$. Let $\mathbb{P}_n = n^{-1} \sum_{i=1}^{n} \delta_{\boldsymbol{y}_i}$ denote the empirical measure where $\delta_{\boldsymbol{y}_i}$ denotes a Dirac measure concentrating on $\boldsymbol{y}_i$. By the definitions of $\hat{\boldsymbol{\eta}}$ and $\boldsymbol{\eta}$ (Equations 13 and 12), we rewrite the

left-hand side of Equation 16 (multiplied by $\sqrt{n}$) as

$$\sqrt{n}\left[\hat{\boldsymbol{\eta}}(\hat{\boldsymbol{\xi}})-\boldsymbol{\eta}(\hat{\boldsymbol{\xi}})\right]=\sqrt{n}\left\{\int \mathbf{H}(\boldsymbol{y};\hat{\boldsymbol{\xi}})\mathbb{P}_n(d\boldsymbol{y})-\int \mathbf{H}(\boldsymbol{y};\hat{\boldsymbol{\xi}})f(\boldsymbol{y};\hat{\boldsymbol{\xi}})d\boldsymbol{y}\right\}$$
$$=\underbrace{\sqrt{n}\left\{\int\left[\mathbf{H}(\boldsymbol{y};\hat{\boldsymbol{\xi}})-\mathbf{H}(\boldsymbol{y};\boldsymbol{\xi})\right]\left[\mathbb{P}_n(d\boldsymbol{y})-f(\boldsymbol{y};\boldsymbol{\xi})d\boldsymbol{y}\right]\right\}}_{\text{(I)}}$$
$$-\underbrace{\sqrt{n}\int \mathbf{H}(\boldsymbol{y};\hat{\boldsymbol{\xi}})\left[f(\boldsymbol{y};\hat{\boldsymbol{\xi}})-f(\boldsymbol{y};\boldsymbol{\xi})\right]d\boldsymbol{y}}_{\text{(II)}}$$
$$+\underbrace{\sqrt{n}\int \mathbf{H}(\boldsymbol{y};\boldsymbol{\xi})\left[\mathbb{P}_n(d\boldsymbol{y})-f(\boldsymbol{y};\boldsymbol{\xi})d\boldsymbol{y}\right]}_{\text{(III)}}. \tag{S4}$$

It suffices to show that

$$\text{(I)}=o_p(1), \tag{S5}$$

and that

$$\text{(II)}=\mathbf{A}(\boldsymbol{\xi})\boldsymbol{\mathcal{I}}^{-1}(\boldsymbol{\xi})\frac{1}{\sqrt{n}}\sum_{i=1}^{n}\nabla_{\boldsymbol{\xi}}\log f(\mathbf{Y}_i;\boldsymbol{\xi})^\top+o_p(1), \tag{S6}$$

in which $\mathbf{A}(\boldsymbol{\xi})=\mathbb{E}\left[\mathbf{H}(\mathbf{Y}_i;\boldsymbol{\xi})\nabla_{\boldsymbol{\xi}}\log f(\mathbf{Y}_i;\boldsymbol{\xi})\right]=\mathrm{Cov}\left[\mathbf{H}(\mathbf{Y}_i;\boldsymbol{\xi}),\nabla_{\boldsymbol{\xi}}\log f(\mathbf{Y}_i;\boldsymbol{\xi})\right]$. Note that $\mathbf{A}(\boldsymbol{\xi})$ is finite because both $\nabla_{\boldsymbol{\xi}}\log f$ and $\mathbf{H}$ are squared integrable in the light of Assumptions (A1) and (A2). Further let $\boldsymbol{\Sigma}_{\mathbf{H}}(\boldsymbol{\xi})=\mathrm{Cov}[\mathbf{H}(\mathbf{Y}_i;\boldsymbol{\xi})]$ and denote column-wise concatenation of matrix blocks by a colon (:). As a consequence of Slutsky's Theorem and the Central Limit Theorem,

$$\sqrt{n}\left[\hat{\boldsymbol{\eta}}(\hat{\boldsymbol{\xi}})-\boldsymbol{\eta}(\hat{\boldsymbol{\xi}})\right]$$
$$=\left[-\mathbf{A}(\boldsymbol{\xi})\boldsymbol{\mathcal{I}}^{-1}(\boldsymbol{\xi})\ :\ \mathbf{I}_{k\times k}\right]\sqrt{n}\begin{bmatrix} n^{-1}\sum_{i=1}^{n}\nabla_{\boldsymbol{\xi}}\log f(\mathbf{Y}_i;\boldsymbol{\xi})^\top \\ n^{-1}\sum_{i=1}^{n}\mathbf{H}(\mathbf{Y}_i;\boldsymbol{\xi})-\boldsymbol{\eta}(\boldsymbol{\xi})\end{bmatrix}+o_p(1)$$
$$\xrightarrow{d}\mathcal{N}\left(\mathbf{0},-\mathbf{A}(\boldsymbol{\xi})\boldsymbol{\mathcal{I}}^{-1}(\boldsymbol{\xi})\mathbf{A}(\boldsymbol{\xi})^\top+\boldsymbol{\Sigma}_{\mathbf{H}}(\boldsymbol{\xi})\right), \tag{S7}$$

which is the desired result (i.e., the right-hand side of Equation 15 with $\boldsymbol{\varphi}=\boldsymbol{\iota}$).

To establish Equation S5, we note that $\sqrt{n}[\mathbb{P}_n(d\boldsymbol{y}) - f(\boldsymbol{y};\boldsymbol{\xi})d\boldsymbol{y}]$ is the empirical process (van der Vaart, 2000, Chapter 19), and that $\mathbf{H}(\cdot;\hat{\boldsymbol{\xi}})$ can be viewed as a random function (of $\boldsymbol{y}$) that estimates $\mathbf{H}(\cdot;\boldsymbol{\xi})$ (as $\hat{\boldsymbol{\xi}} \xrightarrow{p} \boldsymbol{\xi}$ when the model is correctly specified). Then Equation S5 is a straightforward consequence of Lemma 19.24 in van der Vaart (2000) provided (a) $\{\mathbf{H}(\cdot;\boldsymbol{\xi}_1) : \boldsymbol{\xi}_1 \in \mathcal{F}_\omega\}$ is a Donsker class and (b) $\int \left\|\mathbf{H}(\boldsymbol{y};\hat{\boldsymbol{\xi}}) - \mathbf{H}(\boldsymbol{y};\boldsymbol{\xi})\right\|^2 f(\boldsymbol{y};\boldsymbol{\xi})d\boldsymbol{y} \xrightarrow{p} 0$ under the correctly specified model. Both (a) and (b) are guaranteed by Assumption (A2); see Example 19.7 of van der Vaart (2000). To show Equation S6, note that (II) can be further bounded by

$$
\begin{aligned}
\text{(II)} = &\underbrace{\sqrt{n}\int \mathbf{H}(\boldsymbol{y};\hat{\boldsymbol{\xi}})\left[f(\boldsymbol{y};\hat{\boldsymbol{\xi}}) - f(\boldsymbol{y};\boldsymbol{\xi}) - \nabla_{\boldsymbol{\xi}}\log f(\boldsymbol{y};\boldsymbol{\xi})(\hat{\boldsymbol{\xi}} - \boldsymbol{\xi})f(\boldsymbol{y};\boldsymbol{\xi})\right]d\boldsymbol{y}}_{\text{(II.a)}} \\
&+ \underbrace{\sqrt{n}\int \left[\mathbf{H}(\boldsymbol{y};\hat{\boldsymbol{\xi}}) - \mathbf{H}(\boldsymbol{y};\boldsymbol{\xi})\right]\nabla_{\boldsymbol{\xi}}\log f(\boldsymbol{y};\boldsymbol{\xi})(\hat{\boldsymbol{\xi}} - \boldsymbol{\xi})f(\boldsymbol{y};\boldsymbol{\xi})d\boldsymbol{y}}_{\text{(II.b)}} \\
&+ \underbrace{\sqrt{n}\int \mathbf{H}(\boldsymbol{y};\boldsymbol{\xi})\nabla_{\boldsymbol{\xi}}\log f(\boldsymbol{y};\boldsymbol{\xi})(\hat{\boldsymbol{\xi}} - \boldsymbol{\xi})f(\boldsymbol{y};\boldsymbol{\xi})d\boldsymbol{y}}_{\text{(II.c)}}. \qquad \text{(S8)}
\end{aligned}
$$

In Equation S8, (II.a) $= o_p(1)$ is a direct consequence of Assumption (A3) and the $\sqrt{n}$-consistency of $\hat{\boldsymbol{\xi}}$. Moreover,

$$
\|\text{(II.b)}\| \lesssim \sqrt{n}\|\hat{\boldsymbol{\xi}} - \boldsymbol{\xi}\| \left[\int b(\boldsymbol{y})^2 f(\boldsymbol{y};\boldsymbol{\xi})d\boldsymbol{y}\right]^{1/2} \left[\int \|\nabla_{\boldsymbol{\xi}}\log f(\boldsymbol{y};\boldsymbol{\xi})\|^2 f(\boldsymbol{y};\boldsymbol{\xi})d\boldsymbol{y}\right]^{1/2}, \qquad \text{(S9)}
$$

by the Cauchy-Schwarz inequality, in which $x \lesssim y$ means $x \leq Cy$ for some constant C. The squared integrability of $b(\boldsymbol{y})$ and $\nabla_{\boldsymbol{\xi}}\log f(\boldsymbol{y};\boldsymbol{\xi})$ guarantees that the right-hand side of Equation S9 is also $o_p(1)$. Finally, the definition of $\mathbf{A}(\boldsymbol{\xi})$ and Assumption (A1) imply that (II.c) $= \mathbf{A}(\boldsymbol{\xi})\boldsymbol{\mathcal{I}}^{-1}(\boldsymbol{\xi})n^{-1/2}\sum_{i=1}^n \nabla_{\boldsymbol{\xi}}\log f(\mathbf{Y}_i;\boldsymbol{\xi})^\top + o_p(1)$. The proof of Equation S7 is now complete.

A.3 Transformed Residuals $\boldsymbol{e}_{\boldsymbol{\varphi}}$

Let $\boldsymbol{\varphi} : \mathcal{R}^k \to \mathcal{R}^{k'}$, $k' \le k$, be a twice continuously differentiable transformation and $\nabla\boldsymbol{\varphi}$ be the corresponding $k' \times k$ Jacobian matrix. To establish asymptotic normality for the transformed residuals (Equation 14), we Taylor-expand $\boldsymbol{\varphi}\left(\hat{\boldsymbol{\eta}}(\hat{\boldsymbol{\xi}})\right)$ at $\boldsymbol{\eta}(\hat{\boldsymbol{\xi}})$ and obtain

$$\begin{aligned}\sqrt{n}\boldsymbol{e}_{\boldsymbol{\varphi}} &= \sqrt{n}\left[\boldsymbol{\varphi}\left(\hat{\boldsymbol{\eta}}(\hat{\boldsymbol{\xi}})\right) - \boldsymbol{\varphi}\left(\boldsymbol{\eta}(\hat{\boldsymbol{\xi}})\right)\right] \\ &= \sqrt{n}\nabla\boldsymbol{\varphi}\left(\boldsymbol{\eta}(\hat{\boldsymbol{\xi}})\right)\left[\hat{\boldsymbol{\eta}}(\hat{\boldsymbol{\xi}}) - \boldsymbol{\eta}(\hat{\boldsymbol{\xi}})\right] + \mathbf{R}_n. \qquad \text{(S10)}\end{aligned}$$

The remainder vector $\mathbf{R}_n$ can be explicitly expressed as

$$\mathbf{R}_n = \begin{bmatrix} R_{n,1} \\ \vdots \\ R_{n,k'} \end{bmatrix} = \frac{1}{2}\begin{bmatrix} \sqrt{n}\left(\hat{\boldsymbol{\eta}}(\hat{\boldsymbol{\xi}}) - \boldsymbol{\eta}(\hat{\boldsymbol{\xi}})\right)^\top \nabla^2\boldsymbol{\varphi}_{1\cdot}(\boldsymbol{\zeta}_n)\left(\hat{\boldsymbol{\eta}}(\hat{\boldsymbol{\xi}}) - \boldsymbol{\eta}(\hat{\boldsymbol{\xi}})\right) \\ \vdots \\ \sqrt{n}\left(\hat{\boldsymbol{\eta}}(\hat{\boldsymbol{\xi}}) - \boldsymbol{\eta}(\hat{\boldsymbol{\xi}})\right)^\top \nabla^2\boldsymbol{\varphi}_{k'\cdot}(\boldsymbol{\zeta}_n)\left(\hat{\boldsymbol{\eta}}(\hat{\boldsymbol{\xi}}) - \boldsymbol{\eta}(\hat{\boldsymbol{\xi}})\right) \end{bmatrix}. \qquad \text{(S11)}$$

In Equation S11, $\nabla^2\boldsymbol{\varphi}_{i\cdot}, i = 1, \ldots, k'$, denotes the $k \times k$ Jacobian of $\nabla\boldsymbol{\varphi}_{i\cdot}$, which is the transpose of the ith row of $\nabla\boldsymbol{\varphi}$ (i.e., a $k \times 1$ column vector), and $\boldsymbol{\zeta}_n = \gamma\hat{\boldsymbol{\eta}}(\hat{\boldsymbol{\xi}}) + (1-\gamma)\boldsymbol{\eta}(\hat{\boldsymbol{\xi}})$ with some $\gamma \in [0, 1]$. Each component of $\mathbf{R}_n$, i.e., $R_{n,i}$, is $o_p(1)$ because $\sqrt{n}[\hat{\boldsymbol{\eta}}(\hat{\boldsymbol{\xi}}) - \boldsymbol{\eta}(\hat{\boldsymbol{\xi}})] = O_p(1)$ (Equation S7) and $\nabla^2\boldsymbol{\varphi}_{i\cdot}(\boldsymbol{\zeta}_n) \xrightarrow{p} \nabla^2\boldsymbol{\varphi}_{i\cdot}(\boldsymbol{\eta}(\boldsymbol{\xi}))$. By $\nabla\boldsymbol{\varphi}(\boldsymbol{\eta}(\hat{\boldsymbol{\xi}})) \xrightarrow{p} \nabla\boldsymbol{\varphi}(\boldsymbol{\eta}(\boldsymbol{\xi}))$ and Slutsky's Theorem, Equation S10 implies that

$$\sqrt{n}\boldsymbol{e}_{\boldsymbol{\varphi}} \xrightarrow{d} \mathcal{N}\left(\mathbf{0}, \boldsymbol{\Sigma}_{\boldsymbol{\varphi}}(\boldsymbol{\xi})\right), \qquad \text{(S12)}$$

in which $\boldsymbol{\Sigma}_{\boldsymbol{\varphi}}(\boldsymbol{\xi}) = \nabla\boldsymbol{\varphi}(\boldsymbol{\eta}(\boldsymbol{\xi}))\left[-\mathbf{A}(\boldsymbol{\xi})\boldsymbol{\mathcal{I}}^{-1}(\boldsymbol{\xi})\mathbf{A}(\boldsymbol{\xi})^\top + \boldsymbol{\Sigma}_{\mathbf{H}}(\boldsymbol{\xi})\right]\nabla\boldsymbol{\varphi}(\boldsymbol{\eta}(\boldsymbol{\xi}))^\top$ (i.e., Equation 15). For $\boldsymbol{\varphi}(\boldsymbol{\gamma}) = \left(\frac{\gamma_1}{\gamma_{Q+1}}, \ldots, \frac{\gamma_Q}{\gamma_{2Q}}\right)^\top$, where $\boldsymbol{\gamma} = (\gamma_1, \ldots, \gamma_Q, \gamma_{Q+1}, \ldots, \gamma_{2Q})^\top$ as defined in Equation 34, the Jacobian matrix $\nabla\boldsymbol{\varphi}(\boldsymbol{\gamma})$ is a $Q \times 2Q$ matrix given by

$$\nabla\boldsymbol{\varphi}(\boldsymbol{\gamma}) = \left[\operatorname{diag}\left(\gamma_{Q+1}^{-1}, \ldots, \gamma_{2Q}^{-1}\right) : \operatorname{diag}\left(-\frac{\gamma_1}{\gamma_{Q+1}^2}, \ldots, -\frac{\gamma_Q}{\gamma_{2Q}^2}\right)\right], \qquad \text{(S13)}$$

in which diag$(\cdot)$ denotes a diagonal matrix with the given elements on the diagonal.

A.4 Common Factor Models

We proceed to check Assumptions (A1)–(A3) for the common factor model.

Assumption (A1) is implied by standard regularity conditions for the ML estimator, which can be found in many statistical textbooks (e.g., Bickel & Doksum, 2015, Chapter 6; van der Vaart, 2000, Chapter 5). Specifically for mean and covariance structure models of multivariate normal data, which subsumes the common factor model as a special case, readers may refer to, for example, Shapiro (2007) and Magnus and Neudecker (2019, Chapter 15).

Assumption (A2) requires that the function $\mathbf{H}$ is locally Lipschitz continuous in $\boldsymbol{\xi}$ with a squared integrable Lipschitz constant $b(\boldsymbol{y})$ (with respect to $f(\boldsymbol{y};\boldsymbol{\xi})d\boldsymbol{y}$). Under a common factor model, each component of $\mathbf{H}$, which has been expressed in Equation 11 as $H(\boldsymbol{y};\boldsymbol{\xi})$, is in fact continuously differentiable in $\boldsymbol{\xi}$. Hence, we only need to verify that the derivatives of H for all $\boldsymbol{\xi}$ in some $\mathcal{F}_\omega$ are uniformly dominated by an squared integrable function. To this end, note that H can be further expressed as

$$H(\boldsymbol{y};\boldsymbol{\xi}) = h(\boldsymbol{x},\boldsymbol{y};\boldsymbol{\xi})\frac{f(\boldsymbol{y}|\boldsymbol{x})}{f(\boldsymbol{y})} \tag{S14}$$

for the three examples we consider in Section 2.4: $h(\boldsymbol{x},\boldsymbol{y};\boldsymbol{\xi}) = \phi(\boldsymbol{x};\boldsymbol{\xi})$ for the LV normality test, $h(\boldsymbol{x},\boldsymbol{y};\boldsymbol{\xi}) = y_{ij}$ for the MV-level linearity test, and $h(\boldsymbol{x},\boldsymbol{y};\boldsymbol{\xi}) = (y_{ij} - \nu_j - \boldsymbol{\lambda}_j^\top \boldsymbol{x})^2$ for the MV-level homoscedasticity test. When the ratio-form estimator is used for the MV-level tests, the function h in the denominator is simply 1, i.e., $h(\boldsymbol{x},\boldsymbol{y};\boldsymbol{\xi}) = 1$. The $\boldsymbol{\xi}$-derivatives of Equation S14 can then be expressed as

$$\nabla_{\boldsymbol{\xi}} H(\boldsymbol{y};\boldsymbol{\xi}) = \frac{f(\boldsymbol{y}|\boldsymbol{x})}{f(\boldsymbol{y})}\big\{\nabla_{\boldsymbol{\xi}} h(\boldsymbol{x},\boldsymbol{y};\boldsymbol{\xi}) + h(\boldsymbol{x},\boldsymbol{y};\boldsymbol{\xi})\big[\nabla_{\boldsymbol{\xi}} \log f(\boldsymbol{y}|\boldsymbol{x}) - \nabla_{\boldsymbol{\xi}} \log f(\boldsymbol{y})\big]\big\}. \tag{S15}$$

We claim that $\|\nabla_{\boldsymbol{\xi}} H(\boldsymbol{y};\boldsymbol{\xi})\|^2 \leq \|P(\boldsymbol{x},\boldsymbol{y};\boldsymbol{\xi})\|^2$, in which $P(\boldsymbol{x},\boldsymbol{y};\boldsymbol{\xi})$ is a polynomial of $\boldsymbol{y}$ whose coefficients are continuous functions of $\boldsymbol{\xi}$. This claim follows from three observations. First, $f(\boldsymbol{y}|\boldsymbol{x})$ and $f(\boldsymbol{y})$ are densities of multivariate normal distributions that

are log-quadratic in $\boldsymbol{y}$; therefore, $\nabla_{\boldsymbol{\xi}} \log f(\boldsymbol{y}|\boldsymbol{x})$ and $\nabla_{\boldsymbol{\xi}} \log f(\boldsymbol{y})$ are quadratic polynomials of $\boldsymbol{y}$ (e.g., Magnus & Neudecker, 2019, Section 15.3). Second, our three choices of h's and the corresponding $\nabla_{\boldsymbol{\xi}} h$'s are at most quadratic in $\boldsymbol{y}$. Third, $\mathrm{Cov}(\mathbf{Y}_i|\mathbf{X}_i = \boldsymbol{x}) \preceq \mathrm{Cov}(\mathbf{Y}_i)$ under a common factor model (see Equations 7 and 8), and thus the corresponding density ratio $f(\boldsymbol{y}|\boldsymbol{x})/f(\boldsymbol{y})$ is bounded from above. By the facts that multivariate normal distributions have moments of any order and that the bounding polynomial coefficients are continuous in $\boldsymbol{\xi}$, there exists some $\omega > 0$ and integrable $Q(\boldsymbol{x}, \boldsymbol{y})$ such that $\|P(\boldsymbol{x}, \boldsymbol{y}; \boldsymbol{\xi})\|^2 \leq Q(\boldsymbol{x}, \boldsymbol{y})$ for all $\boldsymbol{\xi} \in \mathcal{F}_\omega$, which establishes Assumption (A2).

Assumption (A3) is closely related to the Fréchet differentiability of a probability measure. To verify Assumption (A3), we first obtain the Taylor-series expansion of $f(\boldsymbol{y}; \boldsymbol{\xi}_1)$, where $\boldsymbol{\xi}_1 \in \mathcal{F}_\omega$, at $\boldsymbol{\xi}$:

$$\begin{aligned} f(\boldsymbol{y}; \boldsymbol{\xi}_1) = f(\boldsymbol{y}; \boldsymbol{\xi}) &+ f(\boldsymbol{y}; \boldsymbol{\xi}) \nabla_{\boldsymbol{\xi}} \log f(\boldsymbol{y}; \boldsymbol{\xi})(\boldsymbol{\xi}_1 - \boldsymbol{\xi}) \\ &+ \frac{f(\boldsymbol{y}; \boldsymbol{\zeta})}{2} (\boldsymbol{\xi}_1 - \boldsymbol{\xi})^\top \left[\nabla^2_{\boldsymbol{\xi}\boldsymbol{\xi}} \log f(\boldsymbol{y}; \boldsymbol{\zeta}) + \nabla_{\boldsymbol{\xi}} \log f(\boldsymbol{y}; \boldsymbol{\zeta})^\top \nabla_{\boldsymbol{\xi}} \log f(\boldsymbol{y}; \boldsymbol{\zeta})\right] (\boldsymbol{\xi}_1 - \boldsymbol{\xi}), \end{aligned} \tag{S16}$$

in which $\boldsymbol{\zeta} = \gamma \boldsymbol{\xi}_1 + (1 - \gamma)\boldsymbol{\xi}$ for some $\gamma \in [0, 1]$. Then we have

$$\begin{aligned} &\left\| \int \mathbf{H}(\boldsymbol{y}; \boldsymbol{\xi}_1) \Big[f(\boldsymbol{y}; \boldsymbol{\xi}_1) - f(\boldsymbol{y}; \boldsymbol{\xi}) - \nabla_{\boldsymbol{\xi}} \log f(\boldsymbol{y}; \boldsymbol{\xi})(\boldsymbol{\xi}_1 - \boldsymbol{\xi}) f(\boldsymbol{y}; \boldsymbol{\xi}) \Big] d\boldsymbol{y} \right\| \\ \lesssim &\frac{1}{2} (\boldsymbol{\xi}_1 - \boldsymbol{\xi})^\top \bigg\{ \int \|\mathbf{H}(\boldsymbol{y}; \boldsymbol{\xi}_1)\| \Big[\nabla^2_{\boldsymbol{\xi}\boldsymbol{\xi}} \log f(\boldsymbol{y}; \boldsymbol{\zeta}) \\ &+ \nabla_{\boldsymbol{\xi}} \log f(\boldsymbol{y}; \boldsymbol{\zeta})^\top \nabla_{\boldsymbol{\xi}} \log f(\boldsymbol{y}; \boldsymbol{\zeta}) \Big] f(\boldsymbol{y}; \boldsymbol{\zeta}) d\boldsymbol{y} \bigg\} (\boldsymbol{\xi}_1 - \boldsymbol{\xi}). \end{aligned} \tag{S17}$$

The first and second derivatives of the multivariate normal log-likelihood are quadratic in $\boldsymbol{y}$ (e.g., Magnus & Neudecker, 2019, Section 15.3). From our earlier argument to verify Assumption (A2), we also know that the components of $\mathbf{H}$ considered in the current work are bounded by polynomials in $\boldsymbol{y}$. Therefore, the integral on the right-hand side of

Equation S17 is uniformly bounded for all $\boldsymbol{\xi}_1, \boldsymbol{\zeta} \in \mathcal{F}_\omega$, which further implies that Equation S17 is $o(\omega)$.

B Empirical Data Analysis Using the Simple Estimator

In Section 2.4.2. of the main document, we discussed that, with the target quantity of interest being the conditional expectation $\mathbb{E}(Y_{ij}|\boldsymbol{x}_\ell;\boldsymbol{\xi})$, Equation 41 can be used as a summary quantity to construct the empirical estimator in Equation 42. This approach provides an alternative to forming the ratio-form estimator by taking the ratio of Equation 37 to Equation 38. Similarly, when the conditional variance $\mathrm{Var}(Y_{ij}|\boldsymbol{x}_\ell;\boldsymbol{\xi})$ is of interest, the following summary quantity

$$H_\ell(\mathbf{Y}_i;\boldsymbol{\xi}) = \frac{[Y_{ij} - \mathbb{E}(Y_{ij}|\boldsymbol{x}_\ell;\boldsymbol{\xi})]^2 f(\mathbf{Y}_i|\boldsymbol{x}_\ell;\boldsymbol{\xi})}{f(\mathbf{Y}_i;\boldsymbol{\xi})} \tag{S18}$$

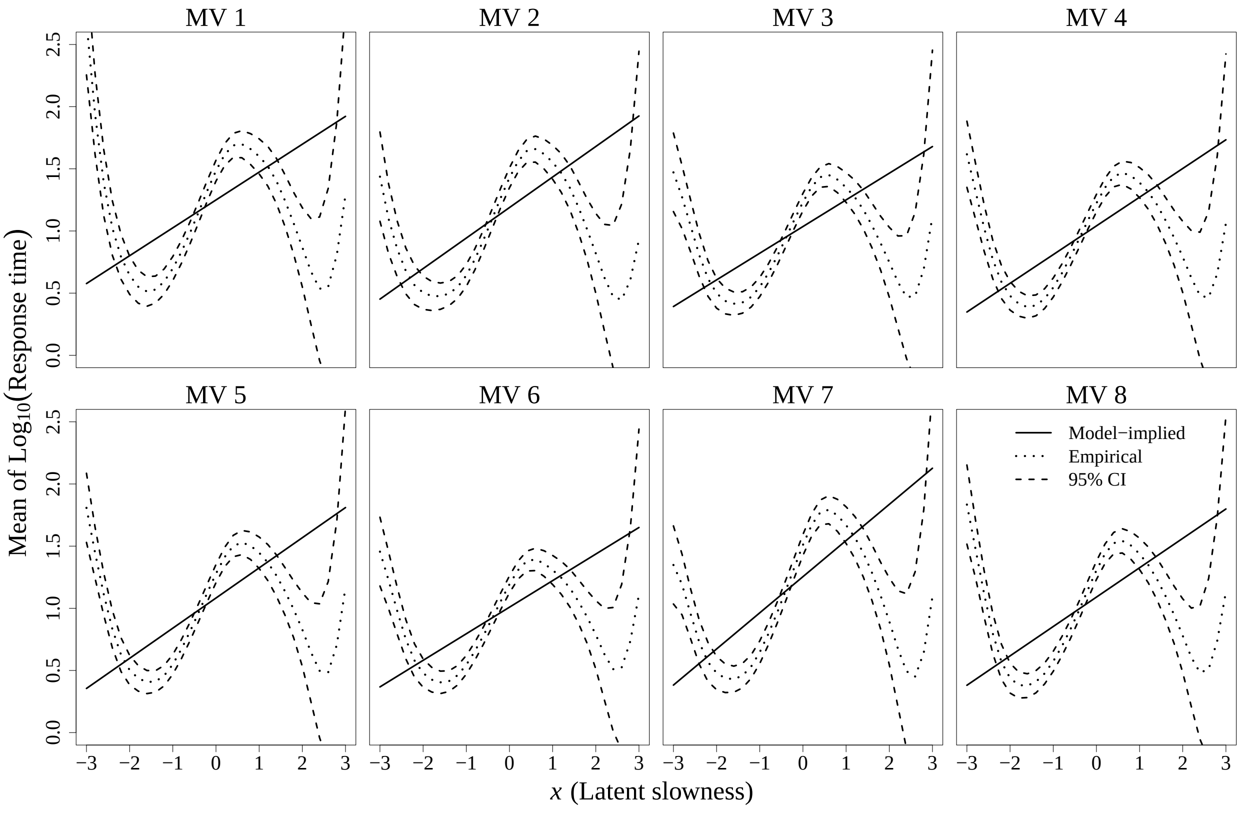

Figure S1: Conditional mean functions of the empirical example analyzed with the simple estimator. Solid lines represent model-implied linear mean functions. Dotted lines represent the simple estimators connected by lines. Dashed lines delineate 95% pointwise confidence bands.

can be used to construct the empirical estimator without taking a ratio. Here, we refer to these alternative versions of empirical estimators, which avoid transformations, as the simple estimator, while the ratio-form estimator in our proposed formulation is referred to as the ratio estimator. The purpose of this section is to highlight the distinctions between these two approaches for MV/item-level fit assessment using the real response time (RT) data presented in Section 4.

When the ratio estimator is used, fit diagnostics are conducted (approximately) independently of the correct specification of the LV density because the ratio estimator estimates the LV density within its formula, as already noted in Section 2.4.2. Although the impact of misfit in LV density cannot be completely eliminated since the estimates of

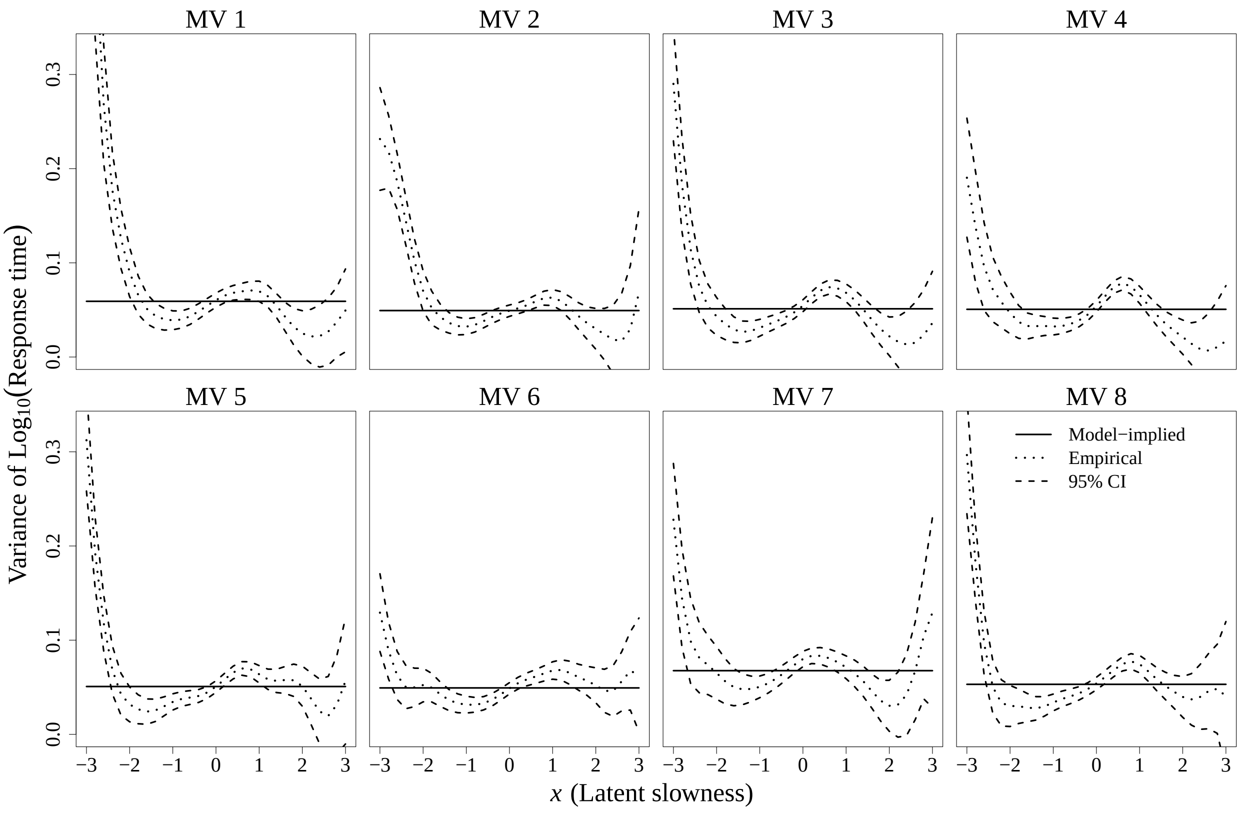

Figure S2: Conditional variance functions of the empirical example analyzed with the simple estimator. Solid lines represent model-implied constant variance functions. Dotted lines represent the simple estimators connected by lines. Dashed lines delineate 95% pointwise confidence bands.

LV density may not perfectly reproduce the true density, the results in Figures 9 and 10 in the main document can still be interpreted as fit diagnostics under the assumption of a correctly specified LV density.

In contrast, using the simple estimator for the same fit assessment would likely lead to all items displaying a similar pattern, due to the influence of a misspecified LV density. Figures S1 and S2 show the item fit assessment results using the simple estimator, where noticeably different shapes are observed compared to those obtained with the ratio estimator. Specifically, the conditional mean and variance functions show a consistent pattern across all items. Although this uniform pattern across all items also provides diagnostic information on badness of fit, this result may not be optimal for the purpose of item-level fit assessment, as it fails to distinguish between well-fitting and poorly-fitting items.

References

Bickel, P., & Doksum, K. (2015). *Mathematical statistics: Basic ideas and selected topics* (2nd ed.). CRC Press.

Magnus, J., & Neudecker, H. (2019). *Matrix differential calculus with applications in statistics and econometrics*. Wiley.

Shapiro, A. (2007). Statistical inference of moment structures. In S.-Y. Lee (Ed.), *Handbook of latent variable and related models* (pp. 229–260). Elsevier.

van der Vaart, A. (2000). *Asymptotic statistics*. Cambridge University Press.
